# A First Step Towards Black Soldier Fly Larvae (Diptera: Stratiomyidae) Welfare by Considering Dietary Regimes (Part I)

**DOI:** 10.3390/insects15100817

**Published:** 2024-10-18

**Authors:** Arianna Cattaneo, Simona Belperio, Luca Sardi, Giovanna Martelli, Eleonora Nannoni, Marco Meneguz, Sihem Dabbou

**Affiliations:** 1 Center Agriculture Food Environment (C3A), University of Trento, 38098 San Michele All’Adige, Italy; arianna.cattaneo@unitn.it; 2 Department of Veterinary Sciences (DIMEVET), University of Bologna, 40064 Ozzano dell’Emilia, Italy; simona.belperio2@unibo.it (S.B.); luca.sardi@unibo.it (L.S.); giovanna.martelli@unibo.it (G.M.); eleonora.nannoni2@unibo.it (E.N.); 3 BEF Biosystems s.r.l., 10156 Turin, Italy; marco.meneguz@bef.bio

**Keywords:** black soldier fly, welfare, scale of rearing, diet composition

## Abstract

The insect farming sector is expanding, but knowledge of insect welfare is still limited. This article aims to optimize the dietary regime for “black soldier fly” (*Hermetia illucens* L., BSF) larvae by applying a holistic view of welfare. Four diets were tested: control (CONTR, commercial laying hen feed), vegetable (VEG), omnivorous (OMN), and carnivorous (MEAT) diet, conducting experiments at a large (2000 larvae) and small scale (100 larvae). Rearing parameters were calculated including the growth rate, substrate reduction, efficiency of conversion of digested food, waste reduction index, and survival rate. Chemical analyses were conducted on BSF larvae and the residual frass. While the MEAT diet appears to be non-well-performing for the larvae, the VEG diet performed comparably to the control diet. Interestingly, the OMN diet demonstrated improved efficiency when evaluating the growth process at both scales. The chemical composition of larvae and frass highlighted the nutritional adequacy of the OMN diet, with the BSF larvae showing adequate protein and lipid content without nutrient catabolism or signs of discomfort. Applying the five freedoms of Brambell’s report as a welfare standard for animal rearing and evaluating performance as an indirect indicator of welfare, the OMN diet appears to promote larval welfare in rearing practices.

## Simple Summary

Rearing management, with a focus on welfare, provides a solid foundation for traditionally farmed animals. In the field of insect rearing, howeverthere is a significant lack of knowledge regarding welfare assessment. This study investigates the larval stage of the “black soldier fly” (Diptera: Stratiomyidae), examining how different dietary regimes impact their welfare. By evaluating performance parameters as indirect indicators of welfare and applying the five freedoms from Brambell’s report, control, vegetable, omnivorous, and meat diets were studied. The finding revealed that an omnivorous diet ensures efficient larval growth without causing movement difficulties or an excessive quantity of dietary fibers. This holds a key relevance in the future development of the insect-rearing sector and for the regulatory approval of new rearing substrates.

## 1. Introduction

Insect bioconversion is one of the most interesting methods of upcycling and creating a circular economy [1]. The species “black soldier fly” (*Hermetia illucens* L.; BSF) (Diptera: Stratiomyidae), with its short life cycle and high feed conversion ratio, can reduce waste biomass by 50–60%, yielding a new material (larval body) rich in high-quality protein [2,3]. According to Regulation (EC) No. 1069/2009, insects fall under the definition of “farmed animals” where they are reared to become animal feed; consequently, the use of certain materials (manure, catering waste, and processed animal protein except fishmeal) for their diet is prohibited [4]. Nevertheless, the International Platform of Insect and Food and Feed stated in 2022 that “exploring the use of new substrates as feed for insects” is a key aspect for the coming years [5]; in fact, incorporating former foodstuffs such as meat and fish, along with food waste, into the insects’ diet could bring about a significant transformation, as it allows for more efficient recycling [5]. Insect welfare is a growing concern due to the future growth of the insect-rearing sector [6,7,8,9,10],in fact as proposed by Cohen et al. [11,12] the human treatment of the insects represent a key point. For traditionally farmed animals, the so-called “5 freedoms” enunciated in “Brambell’s Report” [13] represent welfare guidelines; moreover, the reference of Grandin et al. [14] underlines the need to make measurements for evaluating and ensuring animal welfare.

The European Union is active in welfare assessment for traditionally farmed animals; in fact, the Commission Decision 2017/C 31/12 created a group of experts called ‘Platform on Animal Welfare’ that assists the Commission on topics directly related to animal welfare [15], playing a significant role in the “Farm to Fork Strategy” [16]. In October 2021, the European Commission indicated its future mandates to the European Food Safety Authority (EFSA) concerning animal welfare topics. In this list were indicated “certain invertebrates such as decapods”, but unfortunately no other reference to the insect was present [17]. Research and legislative references are currently missing in the insect sector. Since the year 2019, the International Platform of Insects for Food and Feed (IPIFF), being conscious of the future growth of the insect sector, has recommended the “ethical production of insects” despite the lack of EU legislation on insect welfare [7]. The IPIFF stated that “5 freedoms of Brambell’s report” was the right reference for creating welfare practices for insects [7,18].

Considering BSF, interspecific interactions (pathogens, parasites, and predators), abiotic conditions (temperature, humidity/moisture, substrate aeration, light, pupation substrates, and adult spatial needs), larval and adult nutritional considerations, injury and crowding, handling-associated stress, and slaughter methods impact on their welfare [19]. The literature studied the effect of diet on larval development time, body composition, and weight; however, there is a lack of information regarding their welfare [19].

The present research aimed to identify the most suitable rearing substrate for BSF larvae, representing the initial stage in adopting an insect welfare approach. Larval nutrition is one of the biotic parameters that needs to be considered as related to the welfare status of the larvae. Moreover, the research on nutritional inadequacies for larvae represents a key research area for the coming years [19].

We examined vegetable, carnivorous, and omnivorous diets, due to the future introduction of meat-based substrates as suggested by IPIFF [20]. The main goal was to identify a diet that promotes the welfare of the larvae by enhancing development time, substrate reduction, survival rate, larval weight, and chemical composition, focusing on the adult larval stage with the aim of obtaining valuable animal feed.

Population-level mortality can be used for assessing the subjective welfare of individuals [19]; in this research paper, the focus was on evaluating the performance parameter aiming at applicative research. In fact, performance parameters can decline as welfare indicators since they underline respect for freedom from hunger, thirst, and malnutrition (first freedom); freedom from discomfort (second freedom); and freedom to express normal behavior (fifth freedom), since the larvae were able to move and efficiently degrade the dietary regime, gain weight, and accumulate fat and protein in their bodies. This creates solid knowledge of one of the basic aspects that regulates the insect-breeding process, integrating and ensuring its welfare in industrial production.

## 2. Materials and Methods

### 2.1. Study Site

The study was conducted at “BEF Biosystems” company, located in North Italy (Casalnoceto, AL). The 6-days-old larvae used in this study were derived from their colony. In the company’s laboratory, flying adults were housed in a steel frame cage (100 × 63 × 110 cm) covered with a mosquito net and exposed to LED light. The photoperiod was maintained at 12 h of light and 12 h of dark, as described by Oonincx et al. [21]; the BSFs were maintained in a climate-controlled room with a temperature of 27 ± 1 °C and a relative humidity of 70 ± 5%. Throughout their lifespan, the adult flies had continuous access to water and utilized wooden sticks within the cage for oviposition. These sticks were checked daily and replaced every second day, ensuring the collection of larvae that were uniformly aged, which aided their development throughout the study. The eggs were collected as described by Dortmans et al. [22]: a paintbrush was used to transfer the eggs laid by multiple females from the wooden sticks to plastic cups. The eggs were transferred to the plastic boxes (60 × 40 × 12 cm) used for the hatching phase. Once hatched, the larvae were fed with a mixture of chicken feed and water for 6 days.

Over the years, the company has developed a genetic heritage that has made it possible to obtain individuals with similar performance, resistance, and vitality characteristics.

### 2.2. Rearing Substrates

The diets under analysis were the same as those examined in Belperio et al. [23]; more information is available in Table 1. While Belperio et al. [23] focused on how dietary composition affects temperature development during rearing, this research aimed to evaluate growth performance as an indirect welfare indicator for BSF larvae with the goal of improving their welfare.

The vegetable by-products were obtained from Stroppiana Company (Turin, Italy), while the meat ingredients were produced by Barf Company (Tortona, Italy). For the control diet, the commercial hen feed was provided by Progeo (Reggio Emilia, Italy). The substrates were prepared by mixing and blending each ingredient to reduce their size to below 3 mm using the mixer Katana 20VV (Sirman, Curtarolo (PD), Italy).

Each diet ingredient was carefully weighed and thoroughly mixed for each box to ensure homogeneity throughout. Consequently, all the diet replicates had the same chemical composition.

### 2.3. Experimental Design

**Figure 1:**
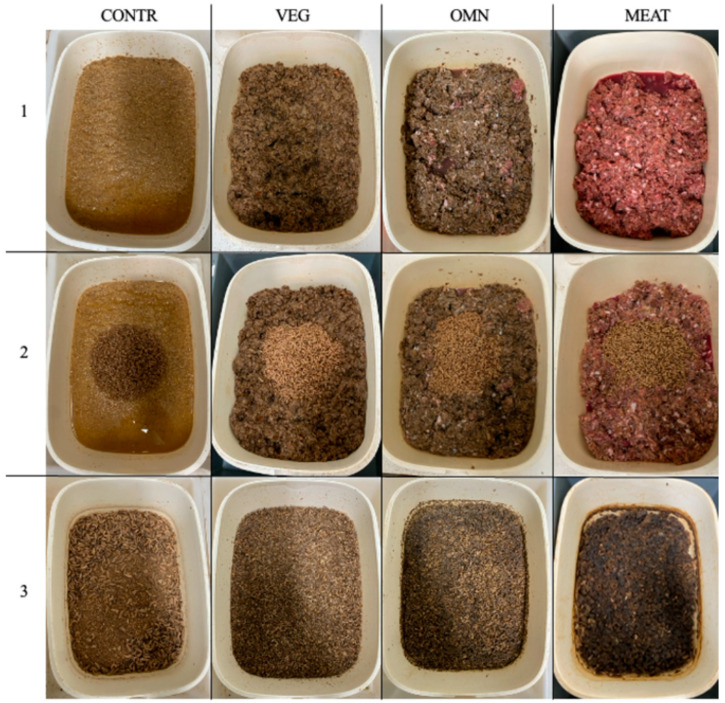
Rearing of black soldier flies on different diets at standard scale (SS) (visible in row 1) The 6-days-old larvae’ sowing (visibile in row 2), end of the trial (visible in row 3). CONTR: commercial feed for laying hens; VEG: 100% vegetable by-products; OMN: 50% vegetable by-products + 50% meat and fish by-products; MEAT: 100% meat and fish by-products.

A homogeneous pool (2 kg) of 6-days-old BSF larvae was separated from their rearing substrate through a vibrating sieve (2 mm, VibroWest MR 24//5.5.5, Milano, Italy). The individual weight of a representative sample (300 larvae) was determined using a digital scale (USS-DBS46-1, U.S. Solid, Cleveland, USA), with the average larval weight equal to 16.67 mg. In parallel, the experiment was conducted on two scales of larval rearing: standard scale (SS) and laboratory scale (LS). Following Bosch et al. [24], four replicates were set up for each treatment, with the trial starting on the same day. This approach allowed us to calculate standard deviations, perform statistical evaluations, and detect any outliers in the data. The 6-days-old larvae were reared in plastic boxes (SS = 32 × 23.5 × 11.5 cm, LS = 8 × 8 × 5 cm). Figure 1 reports some highlights of the rearing process at SS.

For SS, starting from the average larval weight, a group containing an estimated 2000 larvae was created for each treatment. For LS, 100 larvae were manually counted for each treatment. On the standard scale, 2000 g of diet was used as the rearing substrate, while on the laboratory scale, the amount of rearing substrate given to the larvae was 100 g for each treatment following the feeding rate suggested by Dienet et al. [25] as 100 mg feed/larva/day. For each scale of the trial, the boxes were placed on a table according to a 4 × 4 design and shifted one position twice daily to ensure similar environmental conditions during the trial. The boxes were stored at constant temperature and humidity (27 ± 1 °C, 65 ± 5% relative humidity).

Considering day 0 as the moment of the larvae’s sowing, starting from day 2, a ventilator was inserted in the room to obtain the desired drying of the material with an airflow of 1.8 m/s. The lids of the boxes were kept until day 4 to avoid the flies’ contamination due to the summer period. After day 4, the lids were removed for the rest of the experiment, as the BSF larvae had reached an advanced stage of development, which consequently facilitated evaporation and gas exchange. The experiment ended when the materials reached an optimal level of dryness for the sieving phase, which occurred on day 8 for the SS trial and on day 7 for the LS trial, respectively.

The larvae and residual substrate (frass) were separated by processing each box’s content with a 0.6 cm mesh sieve. Subsequently, the insects were killed by freezing at −20 °C to minimize their suffering.

### 2.4. Larval Growth Parameters

At the end of the experiment, the larvae’s biomass and the residual rearing substrate were carefully separated using a vibrating sieve (VibroWest MR 24//5.5.5, Milano, Italy). Each larvae and frass treatments were weighed using a scale (GAB 12K0.1N, KERN Balingen, Germany, maximum 6 kg, d = 0.05 g)) and recorded based on wet weight. For both scales of trial, the following parameters were calculated:

Growth rate (GR) [26,27]:

$$\frac{Average\;final\;larval\;weight\;\left(mg\right)-Average\;initial\;larval\;weight\;(mg)\;}{number\;of\;days\;of\;trial\;(d)}$$

Substrate reduction (SR) [26]:

$$\frac{Initial\;substrate\;weight\left(g\right)-final\;substrate\;weight\;\left(g\right)\;}{initial\;substrate\;weight\;\left(g\right)}\times 100$$

Waste Reduction Index (WRI) [26]:

$$\frac{\frac{Initial\;substrate\;weight\;\left(g\right)-final\;substrate\;weight\;(g)\;}{initial\;substrate\;weight\;(g)}}{number\;of\;days\;of\;trial\;(d)}\times 100$$

Efficiency of conversion of digested food (ECD) (adapted by Leong et al. [26]):

$$\frac{\;Final\;larval\;biomass\;weight\;(g)\;}{Initial\;substrate\;weight\;\left(g\right)-final\;substrate\;weight\;(g)\;}$$

Feed Conversion Ratio (FCR) (adapted by Leong et al. [26]):

$$\frac{Initial\;feed\;biomass\;\left(g\right)-Final\;frass\;biomass\;(g)\;}{Final\;larval\;biomass\;\left(g\right)-Initial\;larval\;biomass\;(g)}$$

Feeding rate (FR) [25]:

$$\left(\frac{Initial\;feed\;biomass\;\left(g\right)}{days\;of\;trial}\right):estimated\;number\;of\;larvae$$

At the end of the experiment, a representative sample of 100 larvae for each replicate was weighed individually using a scale ( USS-DBS46-1, U.S. Solid, Cleveland, OH, USA) to determine the average larval weight.

For the SS farming, a subsample of 10 larvae was analyzed (after freezing) using the ImageJ software (Version 1.53, 2022, National Institutes of Health, Bethesda, MD, USA, determining the average length of the larvae by measuring them from the mouthparts to the lower part of the last abdominal segment. In the LS farming, the survival rate was assessed by counting the larvae and applying the following formula:

Survival rate (SuR):

$$100-(\frac{Initial\;number\;of\;the\;larvae-final\;numbr\;of\;the\;larvae\;}{initial\;number\;of\;the\;larvae})\times 100$$

### 2.5. Chemical Analysis of Substrates, Larvae and Frass

The chemical composition of the dietary substrates is shown in Table 1, as reported in Belperio et al. [23]. The larvae and frass from the standard scale trials, being a representative and quantitatively suitable sample for chemical analysis, were stored at −20 °C to induce larval death and for the maintenance of chemical properties. The larvae and substrates were freeze-dried at −40 °C for four days to remove all the moisture contained in the organic matter.

All the samples were weighed using theKernGAB balance (GAB 12K0.1N, KERN Balingen, Germany, maximum 6 kg, d = 0.05 g), shredded with the Sunbeam Pb International blender(Sunbeam, Boca Raton, Florida, United States) to reduce the size of the product particles, and stored in hermetically sealed plastic containers to avoid external contamination or moisture accumulation. The samples were analyzed by wet chemistry to measure crude protein (CP) (method 976.06, [28]) using a Kjeldahl nitrogen analyzer (Gerhardt Vapodest50, Gerhardt Gmbh, Königswinter, Germany).

For the standard nitrogen-to-protein conversion factor (N-factor of 6.25), the more precise N-factor of 4.67 proposed by Janssen et al. [29] was used to determine the CP. Starch was determined according to the AOAC method 996.11 and ether extract according to the AOAC method 920.39 [28]. Neutral detergent fiber (NDF) was determined by refluxing 0.5 g of the sample for 1 h at boiling temperature in a medium-porosity crucible using a Fibertec 2010 system (Foss Tecator) [30]. Residual nitrogen (ADF) and acid detergent lignin (ADL) were analyzed according to Van Soest et al. [31]. Ash was determined after 3 h of combustion in a muffle furnace at 550 °C (VULCAN 3-550, Dentsply Neytech, Burlington, NJ, USA).

### 2.6. Statistical Analysis

The R software (R core team, version R 4.1.2 2023) with the R-studio interface (R Studio team, version 2023.03, 2023) was used to analyze and interpret the data. First, the linearity of the data was tested. Then, the Shapiro–Wilk test was performed for each dependent variable (larval average weight, larval biomass, residual biomass, growth rate, substrate reduction, larval length, feeding rate, and survival rate, and chemical composition) to check whether they were normally distributed. A *p* > 0.05 indicates a normal distribution of the variables. The variance was tested using the Levene t-test, assuming homogeneity with a *p* > 0.05. With each variable demonstrating linearity, normality, and homogeneity of variance, a one-way ANOVA was conducted. The effect of the treatment on the variable was considered significant with a *p* < 0.05. The Tukey HSD test was applied to determine the calculated multiple comparisons of means at a 95% family-wise confidence level (significant with a *p* < 0.05).

In the cases where the data violated the assumptions of homogeneity of variance and/or normality, a non-parametric statistical analysis was performed. Firstly, the Kruskal–Wallis rank sum test was conducted for each variable, and the effect of the treatment was considered significant when a *p* < 0.05 was observed. As a post hoc test, the Dunn test was performed, indicating a significant difference with *p* ≤ alpha/2 (where alpha = 0.05). 

## 3. Results

### 3.1. Larval Growth Parameters on Standard Scale Rearing

Table 2 illustrates the larvae rearing performances on a standard scale (SS) depending on diet. The larvae reared on the MEAT substrate showed a GR significantly lower than on the OMN diet (4.99 vs. 7.87 mg/day, *p* = 0.008). The VEG diet had a lower amount of final larval biomass compared to MEAT but was comparable to CONTR and OMN.

The comparable SR of the OMN and VEG diets indicated an optimal nutrient balance for larval metabolism and growth, while significant differences occurred comparing the CONTR diet with the OMN diet (82.7% for CONTR vs. 91.0% for OMN, *p* = 0.010), as indicated in Table 2. The WRI showed a significant difference between the OMN and MEAT diets (*p* = 0.010), as well as ECD (*p* = 0.030).

Interestingly, FCR showed comparable results among CONTR, VEG, and OMN, while MEAT was significantly different (*p* = 0.031) from them. Pupation did not exceed 10% and did not occur in all the boxes.

### 3.2. Larval Growth Parameters at Laboratory Scale Rearing

Table 3 reports the rearing efficiency of the BSF larvae grown at a laboratory scale. The OMN treatment presented satisfactory larval weight, significantly higher than the VEG treatment (*p* = 0.004). A similar pattern was observed for the final frass biomass, significantly higher in OMN compared to VEG (*p* = 0.007). The growth rate of OMN was significantly higher than VEG (13.2 vs. 7.58 mg/day, (*p* = 0.003) but similar to MEAT and CONTR (9.41 and 11.4 mg/day, respectively). A significant difference in substrate reduction efficiency was observed with higher efficiency in CONTR than in VEG and MEAT diets (*p* = 0.006). Following the waste reduction index, MEAT and OMN had a significantly higher WRI than VEG. Significant differences in FCR occurred across most of the treatments (*p* < 0.001). The OMN diet showed a statistically lower FCR than VEG and MEAT (*p* < 0.001). No significant difference was observed in the survival rate among all the treatments (*p* < 0.001).

### 3.3. Chemical Composition of Black Soldier Fly Larvae

**Figure 2:**
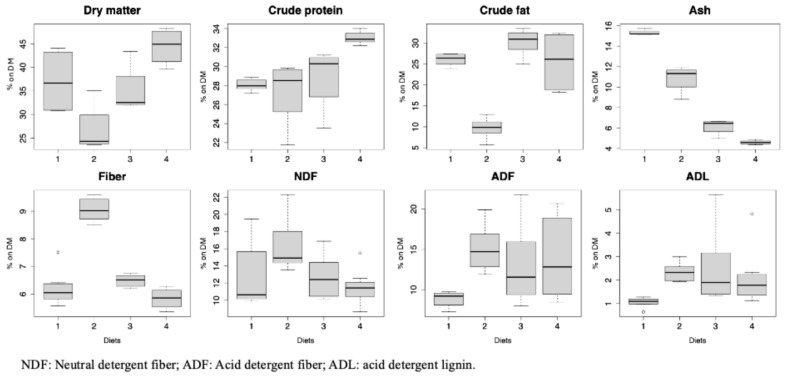
Proximate chemical composition (% on dry matter except where otherwise specified) of black soldier fly larvae reared (mean ± standard deviation; N = 4). 1: CONTR (commercial laying hens’ diet); 2: VEG (100% vegetable by-products); 3: OMN (50% vegetable by-products + 50% meat and fish by-products); 4: MEAT (100% meat and fish by-products).

The chemical composition of the BSF larvae varied significantly depending on the rearing substrate (Figure 2). The MEAT diet had a crude protein content similar to the OMN diet but significantly different compared to CONTR and VEG (*p* < 0.001). As expected, the VEG treatment registered the lowest crude fat content (*p* < 0.001). The fiber content of both the CONTR and MEAT diets was significantly different from VEG (*p* < 0.001), while no differences emerged when comparing OMN and VEG. The dietary treatments showed significant differences in NDF, ADF, and ADL.

The main differences occurred when comparing VEG and MEAT in terms of NDF (*p* = 0.024), as well as CONTR and VEG in terms of ADF (*p* = 0.017).

### 3.4. Chemical Composition of Black Soldier Fly Frass

**Figure 3:**
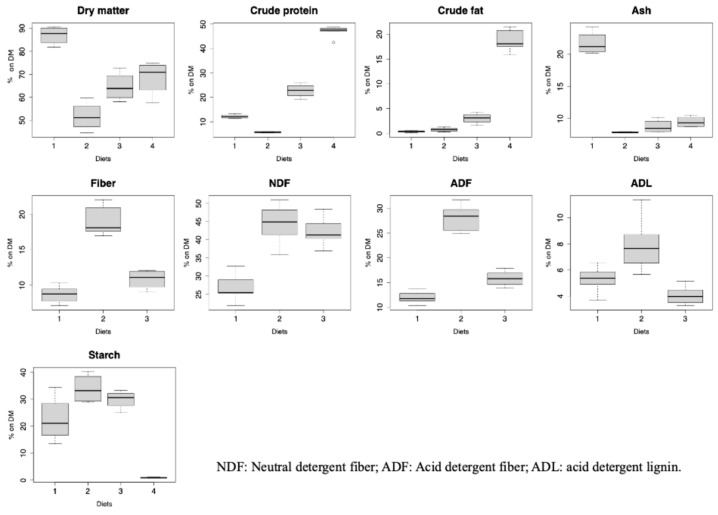
Proximate chemical composition (% of dry matter except where otherwise specified) of black soldier fly frass (mean ± standard deviation; N = 4). 1: CONTR (commercial laying hens’ diet); 2: VEG (100% vegetable by-products); 3: OMN (50% vegetable by-products + 50% meat and fish by-products); 4: MEAT (100% meat and fish by-products).

Figure 3 displays the chemical composition of the black soldier fly frass. All the variables showed significant differences between the groups (*p* < 0.001). The dry matter content in the CONTR frass (86.9%) was significantly different from VEG (*p* < 0.001), but similar to the OMN and MEAT diets. The crude protein content varied significantly across the treatments, with the MEAT protein content at 47.1% DM, and the VEG diet at only 5.77% DM. The crude fat content of the MEAT residual was statistically different from the other treatments (*p* < 0.001), being the higher one.

In terms of ash content, there was a significant difference between the VEG and CONTR treatments (8.72 and 21.7% DM, respectively, *p* < 0.001). The fiber content was also influenced by diet type, with higher contents in the VEG frass followed by the omnivorous and control diets (*p* < 0.001).

Notably, no fiber data were available for the meat substrate. In terms of fiber components, the VEG diet had higher acid detergent fiber and acid detergent lignin contents. The starch content showed significant variability, with significant differences between VEG (33.8% DM) and MEAT (0.85% DM, *p* < 0.001).

## 4. Discussion

To the authors’ knowledge, this is the first study to comprehensively evaluate, through two trials (standard and laboratory scale), the effect of different rearing substrates on the development, waste reduction efficiency, and nutritional composition of BSF larvae while simultaneously using performance parameters as indirect indicators of larval welfare, aiming to identify the most suitable rearing substrate. Research areas that need to be developed for improving the welfare evaluation of insects concern the impact of nutrition and population level on the wellbeing of the larvae [19]. Varying the “population-level mortality” [32] as an evaluation of performance parameters, this study perfectly fits these aims, contributing to the advancement of the insect-rearing sector.

### 4.1. Effect of the Diet on BSF Larvae Performance

The VEG and OMN diets showed satisfactory growth performances in comparison to the CONTR diet. Although the threshold for statistical significance was not reached, the OMN diet was, on average, 10% more efficient in terms of the final average larval weight and 30% more efficient in terms of the final larval biomass compared to the VEG and MEAT treatments, respectively. The growth rate of the OMN larvae was statistically similar to the CONTR and numerically higher than the VEG and MEAT treatments. Interestingly, the substrate reduction in VEG, MEAT, and CONTR was statistically similar.

The laboratory-scale trial highlighted the efficiency of the OMN diet, resulting in a final larval biomass numerically similar to the CONTR. On the contrary, the VEG and MEAT treatments obtained lower larval biomass when compared with CONTR (−30% on average) and elevated frass production (+32% on average, statistically significant for VEG) compared with OMN. The LS trial suggested higher feed conversion efficiency for the OMN and CONTR diets (*p* < 0.001). The VEG and MEAT treatments registered similar performance in terms of FCR. The scientific literature has extensively investigated the nutritional requirements of black soldier fly larvae, as well as the effects of the diet on the insect’s performance [33,34,35,36], but if the purpose is to obtain favorable performances, there is an urgent need to tailor their diet.

For example, the vegetable diets could present some dietary limitations for the larvae [27], suggesting that mixing different types and agro-industrial by-products optimize their performances (development, waste reduction efficiency, and nutritional composition). According to our study, the MEAT diet caused insect metabolization difficulties due to the compact texture [32]. These nutritional imbalances can result in the violations of the first and second freedoms freedom from hunger, thirst, and malnutrition (1), and freedom from discomfort (2) [13].

Looking at the texture of the rearing substrate, the rapid drying of the substrate can cause larval death, but similarly high moisture content or dense textures reduce substrate aeration (due to compromised oxygen diffusion at the surface), creating anaerobic areas dangerous for the larvae [32]. In this sense, the MEAT diet demonstrates significant challenges with regard to substrate aeration and larval mobility. As presented by Gobbi et al. [37], a diet of 100% meat meal registered 60% larval and 80% pupal mortality, and until future discoveries point out processing techniques capable of promoting the survival and welfare of the larva in these substrates, these materials should be avoided for rearing [19]. In this study, the OMN diet was able to improve performance parameters and, therefore, BSF’s overall welfare compared to MEAT and VEG. This result agrees with [34,37], who stated that heterogenous and high-nutrient-content diets with a mix of carbohydrates and proteins increased larval survival. The substrate may be improved in heterogeneity and nutrient composition; mixing them with different materials and maintaining at least 50% nutrient content in any rearing medium is recommended until new discoveries from a welfare perspective [32]. Efficient rearing performances occurred by applying a mix of vegetable and butchery wastes, while using only vegetable by-products the larval production was not satisfying [38].

### 4.2. Standard Scale vs. Laboratory Scale of Rearing

The differences in performance between the two rearing systems found in this study can be well explained by the available literature. BSF larvae tend to stay in contact with each other and, when colonizing a substate, they form “fountains” around the food, attracting new larvae that crawl in from below and are pumped out from above [39,40,41]. Moreover, only a limited number of larvae can feed around the food, as the surface area of the food limits the feeding behavior of the larvae [41]. The surface area available to each larva for movement and feeding was 0.37 cm^2^ in SS and 0.64 cm^2^ in LS; thus, it can be concluded that a larger surface area available to the larvae facilitates their feeding behavior [41].

Furthermore, due to the faster drying of the material, the laboratory scale trails lasted 7 days instead of 8 days, resulting in slightly different feeding rates (SS: 0.12 mg/day/larva; LS: 0.14 mg/day/larva). This difference in feeding rates explains the varying performances between the small- and large-scale trials and supports the hypothesis that the OMN diet is comparable to or even better than the CONTR diet for all the parameters under investigation. In conclusion, considering a general framework, increasing the size of the rearing environment can lead to improved BSF welfare and performances allowing them to move and feed more easily in the substrate, answering the fifth freedom, to express normal behavior [13].

### 4.3. Effects of the Chemical Composition of the Diet on Larvae Performance

#### 4.3.1. Carbohydrate and Protein

A rich protein content characterizes the OMN and MEAT substrates here under analysis. Larval performance is influenced by the total concentration of dietary protein and carbohydrates rather than by the P:C (protein–carbohydrate) ratio, where C is intended as a non-cellulose carbohydrate (NC) [42]. High P + C content improves larval growth, and a diet with a P17:C55 ratio (P:C = 1:3; P + C = 72) guarantees the best larval and adult performance. If we apply the evaluation proposed by Barragan-Fonseca et al. [42] to the OMN substrate (NC: 100 − (CP + CF + Ash + NDF) = 16) the result is a P:C ratio of 1:0.5 and a P + C value of 48.

Further insights were provided by Eggink et al. [43], who tested isoenergetic and isolipidic substrates with different P:C ratios varying between 1:1 and 1:9. The best performance was observed when applying P:C ratios between 1:2 and 1:3. On the contrary, using higher protein levels reduced larval survival and resulted in longer development times [43].

These agree with our results, where a VEG diet characterized by a P:C ratio of 1:10 demonstrated poor larval performance and generally reduced fitness, as confirmed by Fuso et al. [44]. Furthermore, by providing a varied and rich substrate, such as the OMN diet, a P:C of 1:0.5 was sufficient to obtain satisfactory growth results.

Despite the importance of dietary protein content in BSF development, they also have an upper threshold beyond which high CP has a negative effect. In fact, exceeding 37% protein content in the substrate resulted in toxic effects, compromising survival rates and adult emergence [42,45]. In our study, this phenomenon occurred in the MEAT substrates rich in protein, violating the second freedom (from discomfort) as well as third and fifth freedoms (from pain, injury, and disease; to express normal behavior, [13]).

#### 4.3.2. Fat

The MEAT diet was richer in fat because of the origin of the materials, whereas the OMN diet settled at average values. In adult BSF, a diet with a lipid percentage lower or equal to 1% results in negative effects on growth [46]. On the contrary, as highlighted by Ujvari et al. [47], a high fat content in the rearing substrate could induce difficulties in its metabolization.

Interestingly, Liao et al. [48] found that feeding virgin and mated *Drosophila* females with high-saturated fat led to negative effects on their fitness, similar to what happens in humans following the so-called “Western diet.” Additionally, a high-fat diet increased the expression of adipokinetic hormone (Akh), which in turn reduced fitness during chronic high-fat feeding [48,49]. This explains the inadequacy of the MEAT diet from a welfare perspective, as BSF larvae may experience similar negative effects. Notably, the OMN larvae exhibited a high tolerance, even at fat levels of 13.4 g/kg. The analysis of the morphology of the head and buccal apparatus of BSF larvae deserves a mention as it is fundamental to understanding this phenomenon [50]. The larvae’s distinctively robust and conical head wall allows it to easily penetrate the rearing substrate. Furthermore, the mouthparts move in a vertical plane, rotating from a horizontal rest position onwards and downwards, so the insect is capable of grasping particles of semi-liquid food and grinding them into smaller parts with the help of the lacinial teeth [50].

It is, therefore, possible to conclude that larvae find it easier to attack semi-solid substances. In the present study, BSF exhibited good tolerance towards the fat content of the OMN diet since, being of animal origin, it was characterized by fats that tended to be oily in the substrate and easily accessible.

### 4.4. Effect of the Diet on BSF Larvae and Frass Chemical Composition

At the end of the rearing, the OMN larvae showed a high protein content, slightly lower than that of the MEAT larvae. Meanwhile, fat accumulation was predominant in the MEAT-fed larvae. As expected, the larvae fed with the MEAT diet displayed lower levels of ash and fiber.

Studying the frass composition, there were no significant differences between the OMN and MEAT diets. The fiber content, including NDF, ADF, and ADL, was higher in the VEG frass, indicating that BSF larvae had difficulty assimilating these components.

The inclusion of fiber in the diet improves the structure of the substrate, enhancing aeration, movement, feeding, and gas exchange required for BSF growth, ultimately improving the final larval performance [51]. Literature data confirm that BSF can reduce cellulose and hemicellulose levels [48] and exhibits lignocellulolytic potential [52]. However, further investigation is needed to determine if there are upper limits above which BSF larvae cannot operate.

On the other hand, fiber can have a negative impact on the bioconversion process as it is not fully digestible for BSF [53], and this could have led to high fiber residual in the VEG frass. A nutrient imbalance in BSF diets can lead to increased catabolism and excretion, ultimately hindering growth [54]. For instance, the crude protein content of the MEAT frass was notably high, indicating the suboptimal utilization of the nutrients in the substrate. These phenomena can lead to the inefficient use of the available nutrients and a reduction in the fitness of the larvae due to the lack of fiber as a structural agent. From a welfare perspective, the OMN formulation was able to meet the nutritional requirement of BSF without resulting in unused nutrients or stress on the digestion process promoted by enzymatic activity.

Across all the parameters, the statistically significant differences (*p* < 0.001) demonstrate that the type of diet has a profound effect on the frass’s nutrient profile, with each type of diet contributing to significantly different chemical compositions. These data indicate that we can strategically manipulate the nutrient content of BSF frass through diet modification, as well as the wellbeing of the larvae during the rearing.

## 5. Conclusions

Numerous parameters affect the growth of black soldier fly larvae, and among them, the dietary regime can be considered the main factor responsible for an efficient process. This study broadens the scope of insect rearing from a welfare standpoint. It was observed that a diet containing both vegetable and meat ingredients (OMN) contributes to optimal larval growth and welfare, as evidenced by improved performance parameters and larvae chemical composition. This is a crucial step towards the expansion that the insect-farming sector will undergo in the coming years. More research using the experimental design of this study is required to compare results and build stronger evidence in order to further advance knowledge on insect welfare. Moreover, further investigation will explore additional factors (i.e., feeding rate and density) to fully address the welfare requirements of the larvae.

## Figures and Tables

**Table 1:** Formulation and proximate chemical composition (% on dry matter except where otherwise specified) of rearing substrates (control, vegetable, omnivorous, and carnivorous) (when *N* = 2: means ± SD).

Ingredients of Rearing Substrates	CONTR	VEG	OMN	MEAT
	72.72% water, 27.28% laying hens’ feed.	33.33% potatoes, 33.33% carrot, 33.34% beer thresher.	16.66% potatoes, 16.67% carrot, 16.67% beer thresher, 25.00% beef epiglottis, 25.00% cod.	50.00% beef epiglottis 50.00% cod.
Chemical composition				
Dry matter (%)	27.2	30.4	25.6	24.5
Crude protein	14.4	6.61	31.8	49.7
Crude fat	5.35	3.08	13.4	22.2
Ash	13.9	6.41	6.77	6.11
Fiber	5.0^2^	11.8	9.65	n/a
Neutral detergent fiber	17.3	22.6	33.2	n/a
Acid detergent fiber	8.23	19.4	13.9	n/a
Acid detergent lignin	2.73	6.10	4.90	n/a
Starch	33.8	44.5	20.3	2.50

CONTR: commercial feed for laying hens; VEG: 100% vegetable by-products; OMN: 50% vegetable by-products + 50% meat and fish by-products; MEAT: 100% meat and fish by-products; n/a: not available.

**Table 2:** Rearing performance of black soldier fly larvae reared on different substrates (control, vegetable, omnivorous, and carnivorous) at standard scale rearing (2000 larvae; mean ± standard deviation; N = 4).

	CONTR	VEG	OMN	MEAT	*p*
Final larval weight (mg)	79.1 ± 6.35 ^(A)^	72.0 ± 3.96 ^(A,B)^	79.5 ± 1.33 ^(A)^	56.5 ± 4.22 ^(B)^	0.008
Final larval biomass (g)	152 ± 5.7 ^(A)^	142 ± 4.21 ^(A)^	159 ± 4.09 ^(A)^	111 ± 10.11 ^(B)^	<0.001
Final frass biomass (g)	379 ± 26.4 ^(A)^	219 ± 21.6 ^(A,B)^	179 ± 8.99 ^(B)^	346 ± 44.4 ^(A,B)^	0.007
GR (mg/day)	7.82 ± 0.79 ^(A)^	6.94 ± 0.49 ^(A,B)^	7.87 ± 0.16 ^(A)^	4.99 ± 0.53 ^(B)^	0.008
SR (%)	82.7 ± 1.20 ^(B)^	85.4 ± 1.07 ^(A,B)^	91.0 ± 0.44 ^(A)^	82.7 ± 2.22 ^(B)^	0.010
WRI (%)	10.3 ± 0.15 ^(B)^	10.7 ± 0.13 ^(A,B)^	11.4 ± 0.06 ^(A)^	10.3 ± 0.28 ^(B)^	0.010
ECD	0.08 ± 0.00 ^(A,B)^	0.08 ± 0.00 ^(A,B)^	0.08 ± 0.00 ^(A)^	0.07 ± 0.00 ^(B)^	0.030
Larvae length (cm)	1.25 ± 0.08	1.28 ± 0.08	1.35 ± 0.05	1.22 ± 0.1	NS
FCR	15.3 ± 0.68 ^(B)^	15.7 ± 0.66 ^(B)^	14.5 ± 0.52 ^(B)^	21.45 ± 2.74 ^(A)^	0.031
FR (g/larva/day)	0.14 ± 0.00 ^(A)^	0.12 ± 0.00 ^(B)^	0.12 ± 0.00 ^(B)^	0.12 ± 0.00 ^(B)^	0.001

CONTR: commercial feed for laying hens; VEG: 100% vegetable by-products; OMN: 50% vegetable by-products + 50% meat and fish by-products; MEAT: 100% meat and fish by-products; NS: non-significant; GR: growth rate; SR: substrate reduction; WRI: waste reduction index; ECD: efficiency of conversion of digested food; FCR: feed conversion ratio; FR: feeding rate. ^(AB)^ Values with different superscript letters within the same line are significantly different (*p* < 0.05).

**Table 3:** Rearing performance of black soldier fly larvae reared on different substrates (control, vegetable, omnivorous, and carnivorous), at laboratory scale (100 larvae; mean ± standard deviation; N = 4).

	CONTR	VEG	OMN	MEAT	*p*
Final larval weight (mg)	96.9 ± 4.09 ^(A,B)^	69.3 ± 4.41 ^(B)^	108 ± 7.42 ^(A)^	82.8 ± 8.39 ^(A,B)^	0.004
Final larval biomass (g)	10.0 ± 1.10 ^(A)^	6.67 ± 0.35 ^(B)^	10.9 ± 0.39 ^(A)^	6.97 ± 2.35 ^(B)^	0.008
Final frass biomass (g)	20.4 ± 0.49 ^(A,B)^	22.2 ± 0.93 ^(A)^	16.1 ± 0.36 ^(B)^	20.5 ± 0.92 ^(A,B)^	0.007
GR (mg/day)	11.4 ± 0.59 ^(A,B)^	7.58 ± 0.53 ^(B)^	13.2 ± 1.06 ^(A)^	9.41 ± 1.15 ^(A,B)^	0.003
SR (%)	88.2 ± 0.28 ^(A)^	81.4 ± 0.78 ^(B)^	87.6 ± 0.27 ^(A,B)^	81.7 ± 0.36 ^(B)^	0.006
WRI (%)	11.4 ± 0.07 ^(A,B)^	11.1 ± 0.13 ^(B)^	12.0 ± 0.05 ^(A)^	11.3 ± 0.13 ^(A)^	0.012
ECD	0.13 ± 0.01	0.08 ± 0.00	0.13 ± 0.00	0.09 ± 0.02	NS
FCR	9.68 ± 1.30 ^(B,C)^	15.4 ± 1.23 ^(A)^	9.02 ± 0.44 ^(B)^	12.4 ± 0.92 ^(A,C)^	<0.001
FR (g/larva/day)	0.14 ± 0.00	0.14 ± 0.00	0.14 ± 0.00	0.14 ± 0.00	NS
SuR (%)	98.0 ± 1.82	97.0 ± 2.31	98.7 ± 0.50	82.9 ± 29.8	NS

CONTR: commercial feed for laying hens; VEG: 100% vegetable by-products; OMN: 50% vegetable by-products + 50% meat and fish by-products; MEAT: 100% meat and fish by-products; NS: non-significant; GR: growth rate; SR: substrate reduction; WRI: waste reduction index; ECD: efficiency of conversion of digested food; FCR: feed conversion ratio; FR: feeding rate; SuR: survival rate. ^(A–C)^ Values with different superscript letters within the same line are significantly different (*p* < 0.01).

## Data Availability

The data presented in this study are available upon request from the corresponding author.
